# A weighted patient network-based framework for predicting chronic diseases using graph neural networks

**DOI:** 10.1038/s41598-021-01964-2

**Published:** 2021-11-19

**Authors:** Haohui Lu, Shahadat Uddin

**Affiliations:** School of Project Management, Faculty of Engineering, The University of Sydney, 21 Ross St, Forest Lodge, NSW 2037 Australia

**Keywords:** Diseases, Translational research

## Abstract

Chronic disease prediction is a critical task in healthcare. Existing studies fulfil this requirement by employing machine learning techniques based on patient features, but they suffer from high dimensional data problems and a high level of bias. We propose a framework for predicting chronic disease based on Graph Neural Networks (GNNs) to address these issues. We begin by projecting a patient-disease bipartite graph to create a *weighted patient network* (WPN) that extracts the latent relationship among patients. We then use GNN-based techniques to build prediction models. These models use features extracted from WPN to create robust patient representations for chronic disease prediction. We compare the output of GNN-based models to machine learning methods by using cardiovascular disease and chronic pulmonary disease. The results show that our framework enhances the accuracy of chronic disease prediction. The model with attention mechanisms achieves an accuracy of 93.49% for cardiovascular disease prediction and 89.15% for chronic pulmonary disease prediction. Furthermore, the visualisation of the last hidden layers of GNN-based models shows the pattern for the two cohorts, demonstrating the discriminative strength of the framework. The proposed framework can help stakeholders improve health management systems for patients at risk of developing chronic diseases and conditions.

## Introduction

Chronic disease incidence has grown globally, spreading through all regions and affecting all socioeconomic groups^1^. In Australia, for example, the prevalence of chronic diseases has been rising. In the latest report provided by the Australian Institute of Health and Welfare (AIHW), just under half of Australians (47%) had one or more chronic conditions^2^. AIHW reports on ten major chronic condition groups: arthritis, asthma, back pain, cancer, cardiovascular disease, chronic obstructive pulmonary disease, diabetes, chronic kidney disease, mental health conditions and osteoporosis. Nearly 9 in 10 deaths were associated with these chronic diseases in 2018^3^.

Fortunately, the majority of chronic diseases are preventable. The risk of chronic disease can be reduced if at-risk patients were identified early and lifestyle changes were implemented accordingly^1^. Therefore, chronic disease risk prediction plays a key role in healthcare by predicting the patient’s future risk based on their historical medical records. Recently, the accumulation of patient electronic health data, such as electronic health records and administrative claim data, has laid a solid foundation for applying machine learning methods in the medical field, thereby making clinical prediction tasks possible^4^. Using electronic health data to predict chronic diseases does not necessitate any extra time or effort for data collection. Therefore, predicting the risk of chronic diseases using electronic health data and taking prevention steps will dramatically reduce their incidences and related health costs.

A substantial amount of research has been conducted in disease or disaster risk prediction using machine learning and deep learning techniques, as evident in the literature^5–12^. A significant portion of these studies used patient features to train predictive models, including age, gender, behavioural and Body Mass Index. The task was formulated as learning a classifier that infers the prediction outcomes. Meanwhile, using machine learning and deep learning techniques on administrative claim data to generate clinical hypotheses for exploring risk factors offers valuable resources for population health and risk factor discovery^13–15^. Nevertheless, predicting the risk of one chronic disease is complicated by shared risk factors with other comorbidities or conditions. Recently, transforming healthcare data into low-dimensional vectors has become a popular research topic, as it allows machine learning techniques to perform predictive healthcare tasks^16–18^. However, latent relationships exist between chronic diseases and their comorbidities and there are hidden relationships between patients and diseases, which could affect the accuracy of predictions.

To address the problem mentioned above, approaches based on the *Social Network Analysis* (SNA) have lately gained popularity. Researchers proposed SNA approaches to administrative healthcare data to develop networks for different diseases^19^. Khan et al.^20^ used a network-based approach to extract semantics from the *International Classification of Diseases* (ICD) codes that resided in administrative data. They predicted the risk of chronic disease by matching an undiagnosed patient’s health trajectory with the captured network. Lu et al.^21^ constructed a patient network using graph theory and administrative claim data. They used the network features extracted from the patient network in combination with patient features to predict the risk of chronic disease using machine learning methods. However, many of these studies apply basic machine learning algorithms such as logistic regression, random forest and multilayer perceptron artificial neural networks. These existing techniques are somehow computationally expensive. If there is new data input, the corresponding networks need to be redeveloped, and the features from the network need to be recalculated. The GNN-based approach can avoid such recalculations for any new data. To our knowledge, no studies used graph-based deep learning approaches on the administrative claim data in chronic disease prediction.

This study presents a novel weighted graph-based framework for chronic disease prediction using administrative claim data based on the *Graph Neural Network* (GNN). GNN techniques learn node embeddings automatically from the corresponding patient network constructed from the administrative healthcare claim data. Instead of extracting features from the patient network manually, e.g., as in Lu et al.^21^, this proposed framework learns graph’s features at once and uses this information to make predictions. This research has two main goals: first, to model the patients’ latent relationship from a comprehensive weighted patient network, and second, to develop a prediction model using GNN-based techniques by aggregating information directly from the patient network. Given that Cardiovascular Disease (CVD) and Chronic Pulmonary Disease (CPD) are the most common chronic diseases in Australia^22^, they have been chosen as examples of chronic diseases to examine the predictive performance of the framework.

Overall, this paper makes the following two contributions:

- We propose a graph-based view for a group of patients diagnosed with the same disease, named *Weighted Patient Network*, an efficient network method to extract underlying relationships among patients.
- We propose a new framework for predicting the risk of chronic disease based on Graph Neural Networks. We also introduce the GNN-based models into the healthcare research field. This framework can also be implemented for any disease prediction.

The rest of the paper is structured as follows. In “Materials” section, we present the materials include data source, study cohort and features selection for this study. We then present the methodology for disease prediction using Weight Patient Network and GNN in “Methods” section. In “Results” section, we empirically evaluate the proposed framework on disease prediction tasks on real-world administrative claim data. After that, we discuss the framework and highlight some directions for future work in “Discussion” section. Lastly, we conclude our research in “Conclusion” section.

## Materials

The following section describes the data source, selection of the study cohorts and ICD codes range.

### Data source and study cohort

There are two major users of administrative claim data in Australia: the federal government (i.e., Medicare) and private health insurers^23^. The administrative claim data for this research came from the Commonwealth Bank Health Society (CBHS)^24^, an Australian health fund company. It contained the medical histories of around 1,240,000 de-identified patients who received medical services between 1995 and 2018, inclusive. Each medical record for the patient includes a unique patient ID, gender, age, postcode, provider ID, admission, discharge date, claim ID, episode ID, diagnosis procedure code, ICD types and codes and diagnosis-related group codes. The disease codes are defined by the International Classification of Diseases 9th and 10th Australian Modification version (ICD-9-AM and ICD-10-AM)^25^. A series of ICD codes are recorded for each patient’s hospital admission(s) to show what medical conditions the patient had at the time. Like other studies in the literature (e.g.,^20,21^), to build the framework, we are also interested in the information from the patients, such as age, gender, and disease codes.

In this study, we use CVD and CPD as chronic diseases to test the proposed framework. Literature suggests coronary heart disease, cerebrovascular disease, rheumatic heart disease, and other heart and blood vessel disorders are classified as CVDs^26^. We utilise ICD codes to identify patients with CVD, including congestive heart failure, cardiac arrhythmias, valvular disease, pulmonary circulation disorders, and peripheral vascular disorders. The ICD codes for these diseases has been adapted from Quan et al.^27^. Several filtering criteria were applied to the original dataset. The criteria for filtering strategy include: (i) Select patients having at least two admission episodes, as we cannot examine transitions across comorbidities without two consecutive admissions, (ii) Select episodes with related ICD codes, as mentioned in Table 1, and (iii) The maximum admission is set to 50. Some patients may need to be admitted on a regular basis for continuous treatments. These recurrent admissions are not linked to specific diagnoses or illnesses, but rather to a treatment plan for a single underlying problem.

**Table 1:** ICD-9-AM and ICD-10-AM codes for cardiovascular disease (CVD) and chronic pulmonary disease (CPD).

	CVD	CPD
ICD-9-AM codes	398.91, 402.11, 402.91, 404.11, 404.13, 404.91, 404.93, 428.x, 426.10, 426.11, 426.13, 426.2–426.53, 426.6–426.28, 427.0, 427.2, 427.31, 427.60, 427.9, 785.0, V45.0, V53.3, 093.2, 394.0–397.1, 424.0–424.91, 746.3–746.6, V42.2, V43.3, 416.x, 417.9, 440.x, 441.2, 441.4, 441.7, 441.9, 443.1–443.9, 447.1, 557.1, 557.9, V43.4	416.8, 416.9, 490.x–505.x, 506.4, 508.1, 508.9
ICD-10-AM codes	I09.9, I1.0, I13.0, I13.2, I25.5, I42.0, I42.5–I42.9, 143.x, 150.x, P29.0, I44.1–I44.3, I45.6, I45.9, I47.x, R00.0, R00.1, R00.8, T82.1, Z45.0, Z95.0, A52.0, I05.x–108.x, I09.1, I09.8, I34.x–I39.x, Q23.0–Q23.3, Z95.2–Z95.4, I26.x, I27.x, I28.0, I28.8, I28.9, I70.x, I71.x, I73.1, I73.8, I73.9, I77.1, I79.0, I79.2, K55.1, K55.8, K55.9, Z95.8, Z95.9	I27.8, I27.9, J40.x–J47.x, J60.x–J67.x, J68.4, J70.1, J70.3

To predict the risk of CVD and CPD, we choose two cohorts for each disease: CVD patients and non-CVD patients, and CPD patients and non-CPD patients. For non-CVD or non-CPD cohorts, we select patients with at least two admission episodes. For these two groups, we chose patients who did not have any ICD codes used to define CVD or CPD cohort. After this initial selection process, we applied approaches for detecting outliers.

### ICD code grouping

There are more than 20,000 unique and active ICD codes for each format in the administrative data^28^. The analysis and visualisation of ICD codes involve a high level of complexity. Due to this, we filter out disease codes that are not related to chronic diseases or their comorbidities. There are several common lists of comorbidity indices in the literature, such as Charlson^29^ and Elixhauser index^30^. In this study, we choose the Elixhauser index to generate the disease list. In addition, we chose the behavioural feature of *smoking* since it is a major cause of chronic illness and death^31^. We grouped ICD-9 codes "3051", "64900", "64901", "64902"," 64903", "64904", "V1582" and ICD-10 codes "F17", "F17.*", "T65.2", "P04.2", "Z72.0", "Z86.43", "Z58.7" for deciding for a patient’s smoking attribute since they are related to smoking^32^.

### Patient features

According to previous studies, age, gender and smoking history are one of the significant risk factors for chronic diseases^33–35^. Therefore, these three features are considered as node features in the proposed model. After the normalisation, the age risk factor has been converted to a continuous score ranging from 0 to 1. The gender risk factor is a categorical score that does not require any further normalisation: 0 for females and 1 for males. The smoking risk factor has a discrete value of 0 for non-smokers and 1 for smokers.

## Methods

The methods for creating a graph-based view of patients (i.e., *Weighted Patient Network*) are discussed first in this section. This network is meant to show the interaction between patients who have a chronic disease(s) in common. In our case, the disease would be CVD or CPD. There will also be a discussion of a GNN-based methodology for learning graph structure.

### Weighted patient network

**Figure 1:**
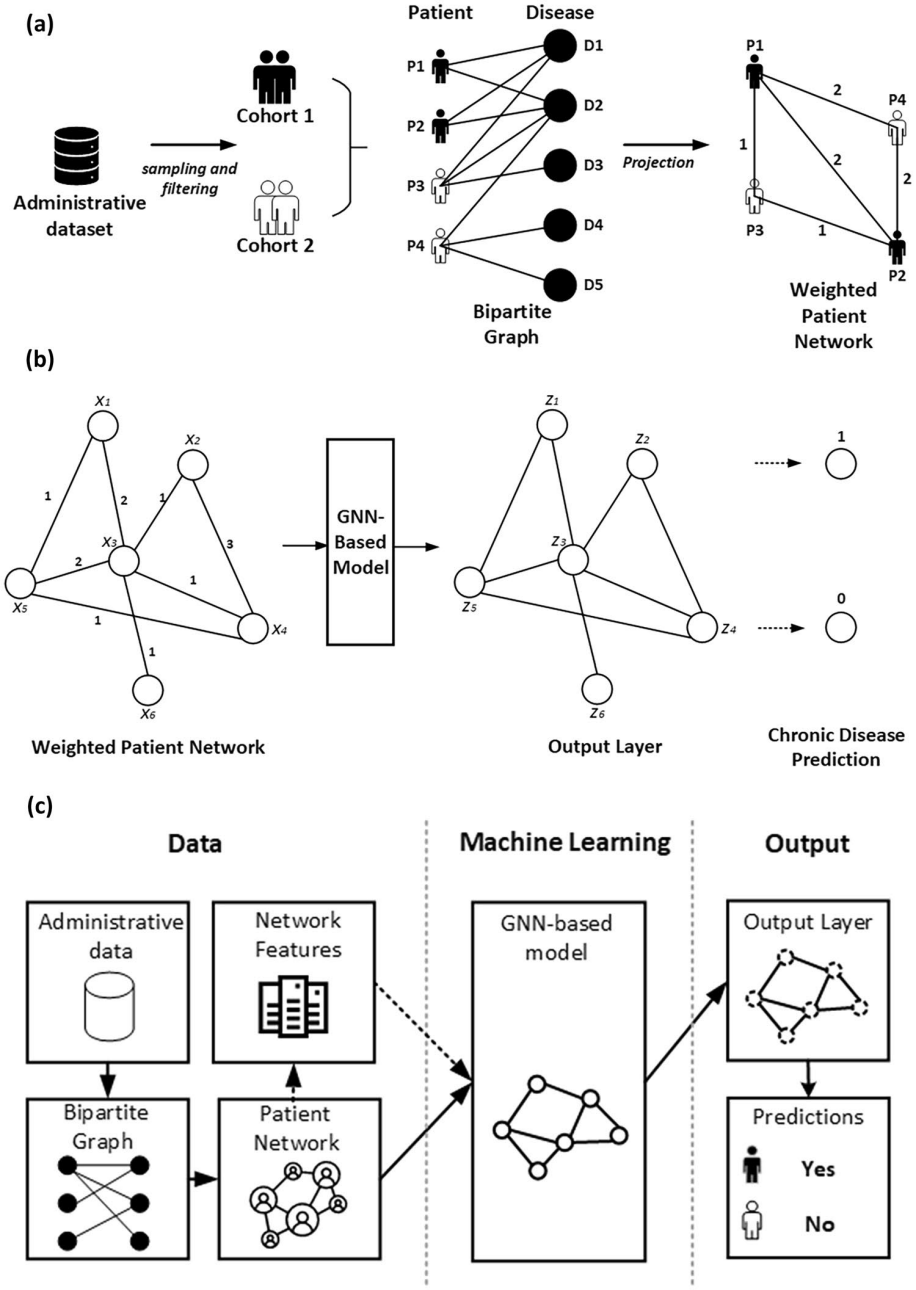
(**a**) Illustration of the process for constructing a weighted patient network. (**b**) The workflow of graph neural network (GNN)-based models for disease prediction. X_1_, X_2_, …, X_n_ are the input features, and Z_1_, Z_2_, …, Z_n_ are the output of the last layer in the GNN-based model. (**c**) Block diagram of the proposed GNN-based framework.

This section briefly describes the method to construct the patient network with edge weight. The weighted patient network has been constructed using the concepts and measures from the graph theory. A bipartite graph is a particular type of graph in the graph theory with two disjoint vertex sets^36^. An undirected bipartite graph is used in this study to show the relationship between the patient and disease. Projecting a bipartite graph onto one of its nodes is a task that has been found useful for further in-depth analyses^21^. We projected the bipartite graph into the ‘patient’ side, named as the ‘*Weighted Patient Network*’ (WPN). Patients are connected in the projected WPN graph by a tie if they are diagnosed with the same disease. The weights between patients are the number of common neighbours. For example, as illustrated in Fig. 1a, patient *P1* has been diagnosed with two common diseases (*D1* and *D2*) with patient *P2*. Therefore, the weight between *P1* and *P2* is 2. Similarly, patient *P1* has been diagnosed with one common disease (*D2*) with *P4*, resulting in a weight of 1 between them. The nodes keep their properties and are connected in the generated graph if they have an edge to a common node in the original bipartite graph. The literature points out that the comorbidity patterns were confirmed by a shared molecular mechanism using disease-gene interactions^37^. Furthermore, evidence shows patients with the same chronic diseases have common risk factors, such as tobacco smoking history, obesity and inadequate physical activity^38^. Therefore, we use WPN to extract latent relationships among patients.

### A GNN-based technique for learning graph structure

To learn the latent relationship between patients and predict the risk of developing the chronic disease for a patient with specific comorbidities, we aggregate neighbourhood information of each patient node from the WPN using the GNN-based framework. For performance analysis and comparison, we use two GNNs variants: Graph Convolutional Network (GCN) and Graph Attention Network (GAT).

GCN is a multilayer connected neural network architecture used to learn low-dimensional node representations from graph-structured data^39^. Through direct graph links, each layer of GCN aggregates neighbouring information to reconstruct embeddings as inputs to the following layer. In particular, given a network and the corresponding adjacency matrix *A*, the layer-wise propagation rule of GCN is defined as follows:

1 $$\begin{array}{*{20}c} {H^{{\left( {l + 1} \right)}} = f\left( {H^{\left( l \right)} ,A} \right) = \sigma \left( {\tilde{D}^{{ - \frac{1}{2}}} \tilde{A}\tilde{D}^{{ - \frac{1}{2}}} H^{\left( l \right)} W^{\left( l \right)} } \right)} \\ \end{array}$$

where *H*^(*l*)^ denotes the embedding of nodes at the *l*^th^ layer, $\tilde{A}$ is the adjacency matrix for added self-connections. $\tilde{D}$ is the diagonal node degree matrix of $\tilde{A}$. *W*^(*l*)^ is a layer-specific trainable weight matrix, and *σ*(·) is an activation function, e.g., the Rectified Linear Unit (ReLU), which gives a non-negative outcome by considering the positive part of its argument^40^.

GAT incorporates the attention mechanism into the propagation steps^41^. It follows the self-attention strategy, and each node’s hidden state is computed by attending over its neighbours. The layer computes the coefficients in the attention mechanism of a node pair (*u* to *v*) using the following formula:

2 $$\begin{array}{*{20}c} {\alpha_{{\left( {u,v} \right)}} = \frac{{\exp \left( {LeakyReLU\left( {a^{T} \left[ {Wh_{u} \parallel Wh_{v} } \right]} \right)} \right)}}{{\mathop \sum \nolimits_{{k \in N_{u} }} \exp (LeakyReLU(a^{T} \left[ {Wh_{u} \parallel Wh_{v} } \right]))}}} \\ \end{array}$$

where *N*_*u*_ is the neighbourhoods of node *u* in the graph, $h = \left\{ {h_{1} , h_{2} , \ldots ,h_{N} } \right\}$ is the input node features, *a*^*T*^ denotes transposition of the weight vector, *W* is the trainable weight matrix of a shared linear transformation and || is the concatenation operation.

Extending the attention mechanism to employ multi-head attention has proven to be advantageous in stabilising the learning process of self-attention. Therefore, *K* independent attention mechanisms are applied to compute the hidden states, and then concatenates or averages (for the last layer) their features^42^, resulting in the following two output representations:

3 $$\begin{array}{*{20}c} {h_{i}^{\prime } = \parallel_{k = 1}^{K} \sigma \left( {\mathop \sum \limits_{{v \in N_{i} }} \alpha_{uv}^{k} W^{k} h_{v} } \right)} \\ \end{array}$$

4 $$\begin{array}{*{20}c} {h_{i}^{\prime } = \sigma \left( {\frac{1}{K}\mathop \sum \limits_{K = 1} \mathop \sum \limits_{{v \in N_{i} }} \alpha_{uv}^{k} W^{k} h_{v} } \right)} \\ \end{array}$$

where $\alpha_{uv}^{k}$ is normalised attention coefficient computed by the *k*th attention mechanism.

We predict the risk of a chronic disease using the learned embeddings from the GNN-based models. Since the label is binary (i.e., if the patient is progressing to CVD, then 1; otherwise 0), the binary cross-entropy loss function is used. This loss function can be optimised via the Adam optimiser^42^. In addition, instead of a binary adjacency matrix, we also use a weighted adjacency matrix with edge weights to train the models. The workflow of our GNN-based model for disease prediction is presented in Fig. 1b.

## Summary of the proposed framework

The input to the proposed framework is the administrative data provided by a private health fund in Australia. Firstly, two study cohorts were created following the filtering and sampling processes (i.e., CVD and Non-CVD, and CPD and Non-CPD). Secondly, a bipartite graph is created, and we used the bipartite projection technique to create a WPN. Then, an edge list is created from this WPN, and we used patient features together to train and test the GNN-based models to predict the chronic disease. Figure 1c shows the block diagram of the proposed framework, and the pseudo-code is presented in Algorithm 1.
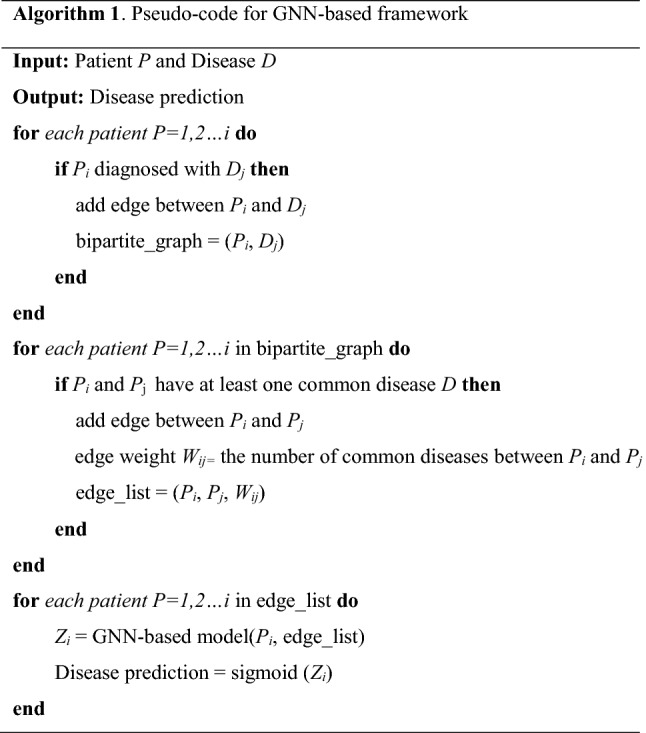


### Statements related to data availability and ethical consideration

- This study obtained research data from an Australian private health insurance organisation (Commonwealth Bank Health Society, CBHS). This data was collected in a de-identified format and through a research agreement between the CBHS and the University of Sydney (University of Sydney reference number: CT18435). For reproducing the results of this study, the relevant data of the study variables can be shared upon request.
- Ethical approval is not required for this study since the Australian legislation permits the retrospective study of de-identified data.
- Under Australian legislation, informed consent is not required for the use of de-identified health insurance data in research.

## Results

This section presents detailed data pre-processing, experimental analysis and evaluations of our proposed disease prediction model.

### Network statistics for CVD and CPD cohorts

In the experiments, we utilise the CBHS dataset to construct the patient network. After applying filtering and data pre-processing techniques, we obtain 1305 patients for the CVD cohort and 528 patients for the CPD cohort. We then select an equal number of non-CVD patients and non-CPD patients at random from the remaining eligible patient list to address the class imbalance problem. These cohorts are filtered using the procedures outlined in “Data source and study cohort” section. We only include records with sufficient information in the form of disease codes. Thus, we consider 2610 patients for the CVD/non-CVD example and 1056 patients for the CPD/non-CPD example to generate the WPN. Table 2 summarises the characteristics of the patient networks. Since some patients do not have a common ICD code, the number of nodes in the patient network is slightly less than the total number of selected patients. The edge counts for CVD and CPD are 138,108 and 31,174, respectively, indicating that the patients are suffering from common diseases in the weighted patient networks. The average degree is the average number of edges per node in the patient network. The average connection for patients in CVD and CPD is 108.873 and 63.041, respectively.

**Table 2:** Characteristics of the patient network.

Characteristics	WPN for CVD	WPN for CPD
Number of nodes	2537	989
Number of edges	138,108	31,174
Average degree	108,875	63.041

### Experimental settings

We randomly divide the dataset into training, validation and test sets in a 0.60: 0.20: 0.20 ratio to develop risk prediction models (i.e., we use 60% of nodes to train the models, 20% of nodes for performance validation and mask 20% of nodes in WPN for testing during training processes). The machine learning models were trained using Python and the Scikit-learn (sklearn) package^43^, while the graph-based models were trained using StellarGraph^44^. There are several hyperparameters in GCN and GAT models, such as the number of layers, the learning rate of the optimiser, the total training epochs and dropout. Further, there are different designs for the GNN architecture based on different factors, such as the implementation of batch normalisation, different type of activation functions and different layers type. We have considered various combinations of these parameters and designs. By adjusting the parameters empirically, we trained all models for a maximum of 1000 epochs using Adam optimiser^42^ with a learning rate of 0.01 and early stopping with the patience of 30 epochs, i.e., the number of epochs to wait before stopping if no further improvement is made. Further, as like Srivastava et al.^45^, dropout with *p* = 0.3 is applied to all layers to prevent overfitting. In addition to these settings, we applied a three-layer model, used a hidden size of 16 units and ReLU activation function for hidden layers in GCN architecture and followed by a sigmoid activation function for classification. We applied a two-layer model for GAT. The first layer consists of *K* = 8 attention heads computing *F* = 8 feature each. After that, it is followed by an exponential linear unit^46^ as an activation function. The second or output layer is used for classification, followed by a sigmoid activation, and a single attention head computes binary class: CVD or non-CVD, and CPD or non-CPD.

### Baseline methods

Our GNN-based models are compared to three well-known classifiers, namely Logistic Regression (LR), Support Vector Machine (SVM), and Random Forest (RF), as well as one deep learning model: artificial neural network (ANN).

**LR** is the method of modelling the probability of a discrete result given an input^47^. **SVM** finds a hyper-plane that separates the different types of data^48^. **RF** combines the output of multiple decision trees to reach a single outcome^49^. **ANN** is a fully connected neural network consisting of a sequence of fully linked layers that connect every neuron in one layer to each neuron in the next layer. Weights and biases are assigned to nodes and edges. These weights and biases can be modified by backpropagating the loss function. The outputs of nodes in the last layer can classify or predict test data based on ANN training^50,51^. For these machine learning classifiers, we employ two different sets of features: (i) patient features only and (ii) network features inspired by previous research^21^. We use degree centrality, eigenvector centrality with weight, closeness centrality, betweenness centrality with weight, and clustering coefficient with weight combined with patient features to predict the risk of chronic disease. We also applied hyperparameter tuning to find the best performance for the baseline methods considered in this study.

### Findings and evaluations

The findings of our comparative evaluation experiments are summarised in Tables 3 and 4.

**Table 3:** Performance of models based on CVD test data. TP, TN, FP and FN stand for True Positive, True Negative, False Positive and False Negative, respectively.

Features	Method	Accuracy (%)	Precision (%)	Recall (%)	F1 (%)	TP	TN	FP	FN
With network features	LR	76.25	76.5	76.28	76.2	210	188	75	49
	SVM	77.97	78.5	77.97	77.88	219	188	75	40
	RF	86.59	86.69	86.59	86.58	217	235	28	42
	ANN	85.06	85.8	85.06	84.97	201	243	20	58
	GCN	90.80	90.85	90.80	90.80	239	235	28	20
	GAT	92.34	92.70	92.34	92.32	251	231	32	8
Without network features	LR	71.26	71.54	71.26	71.19	198	174	89	61
	SVM	67.82	69.74	67.82	67.08	215	139	124	44
	RF	65.52	65.62	65.52	65.52	171	171	92	88
	ANN	71.84	71.97	71.84	71.82	195	180	83	64
	GCN	90.04	90.05	90.04	90.04	231	239	24	28
	GAT	93.49	93.86	93.49	93.48	254	234	29	5

**Table 4:** Performance of models based on CPD test data. TP, TN, FP and FN stand for True Positive, True Negative, False Positive and False Negative, respectively.

Features	Method	Accuracy (%)	Precision (%)	Recall (%)	F1 (%)	TP	TN	FP	FN
With network features	LR	66.98	67.23	66.98	67.05	79	63	32	38
	SVM	66.04	66.06	66.04	65.16	93	47	48	24
	RF	76.42	77.39	76.42	76.47	84	78	17	33
	ANN	72.17	72.14	72.17	72.16	88	65	30	29
	GCN	85.38	86.57	85.38	85.07	112	69	26	5
	GAT	89.15	89.47	89.15	89.06	111	78	17	6
Without network features	LR	58.96	63.77	58.96	57.59	48	77	18	69
	SVM	61.32	60.95	61.32	60.32	88	42	53	29
	RF	60.38	60.98	60.38	60.48	69	59	36	48
	ANN	61.79	61.47	61.79	60.72	83	48	47	34
	GCN	87.26	88.31	87.26	87.03	113	72	23	4
	GAT	89.15	90.04	89.15	88.99	114	75	20	3

A comparison of the accuracy column of Tables 3 and 4 indicates that network features help improve the accuracy for each of the baseline models for both CVD and CPD. For example, the LR accuracy for the CPD data is 63.77% without the network features. This accuracy amount has been increased to 66.98% when network features were considered. Further, graph-based deep learning models (GCN and GAT), outperform baselines by a significant margin, both for with and without network features. This verifies the graph-based model’s effectiveness in representing a patient by aggregating the learned representations from its neighbour nodes. Simultaneously, we discover that while the network features improve the performance of baseline models, there is an insignificant improvement for GNN-based models if we implement network features. This indicates GNN-based models generated node embeddings based on local network neighbourhoods and learned graph representation successfully during the training process. Node embeddings based on complex network features (e.g., betweenness centrality) do not affect their perceived performance. Furthermore, the best accuracy performance is achieved when GAT is applied. GAT achieved an accuracy of 93.49% and 89.15% for CVD and CPD, respectively.

Tables 3 and 4 further show that the outcome for other performance measures for CVD and CPD, respectively. GAT reveals the highest Precision, Recall and F1 among the models for CVD and CPD cases, followed by GCN, indicating the superiority of GNN-based models in predicting chronic disease. In addition, the false-positive count is higher than the false-negative count for the best performed GAT model for two cases. For the proposed framework, this is ideal. Although some amount of clinical resources may be wasted due to these false-positives, a higher number of false-negatives in the prediction will make it unlikely to leave patients who are on the chronic disease pathway undetected. From a population health perspective, it is safer to flag patients, who are not at risk, as chronic-risked (i.e., false positive) than to flag in the opposite direction.

**Figure 2:**
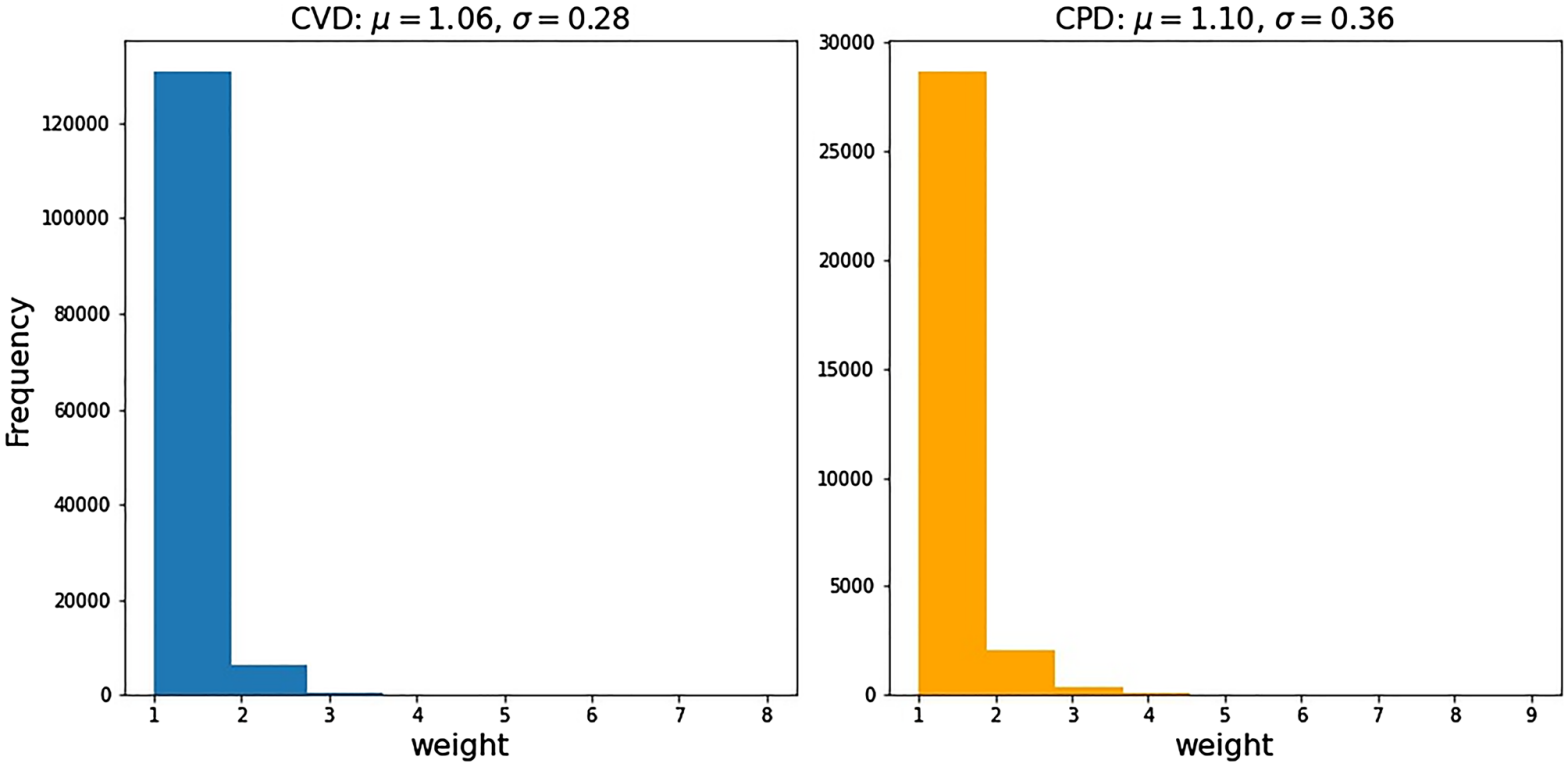
The distribution of edge weight for CVD and CPD.

In order to further evaluate our framework, we train the baseline models and GNN-based models without edge weight in the patient network. Table 5 presents the accuracy measure of the models for CVD and CPD with the patient network without edge weight. Compared to Tables 3 and 4, the performances of LR and SVM for both diseases have been increased, which is opposite to our assumption. We speculate that the network features could not capture the edge weight information from the WPN for these models. Meanwhile, the accuracy of GCN models without edge weight and network features decreases for both diseases, while the best-performed GAT model revealed the same performance as with the edge weight consideration in WPN (in Tables 3 and 4). Figure 2 illustrates the distribution of edge weight in both diseases. Since the majority of the edge weight is 1, the improvement is not significant from the patient network without edge weight to WPN. The network architecture to distinguish the edge weight will require more domain knowledge about the dataset and left for future research.

**Table 5:** The accuracy measure of different models for CVD and CPD without the consideration of edge weight in the patient network.

Features	Method	Accuracy for CVD (%)	Accuracy for CPD (%)
With network features (i.e., degree centrality, eigenvector centrality etc.)	LR	76.63	69.34
	SVM	79.89	66.51
	RF	86.59	79.25
	ANN	83.91	73.11
	GCN	87.26	85.85
	GAT	89.15	88.21
Without network features	GCN	89.08	83.02
	GAT	93.49	89.15

### Node embeddings

**Figure 3:**
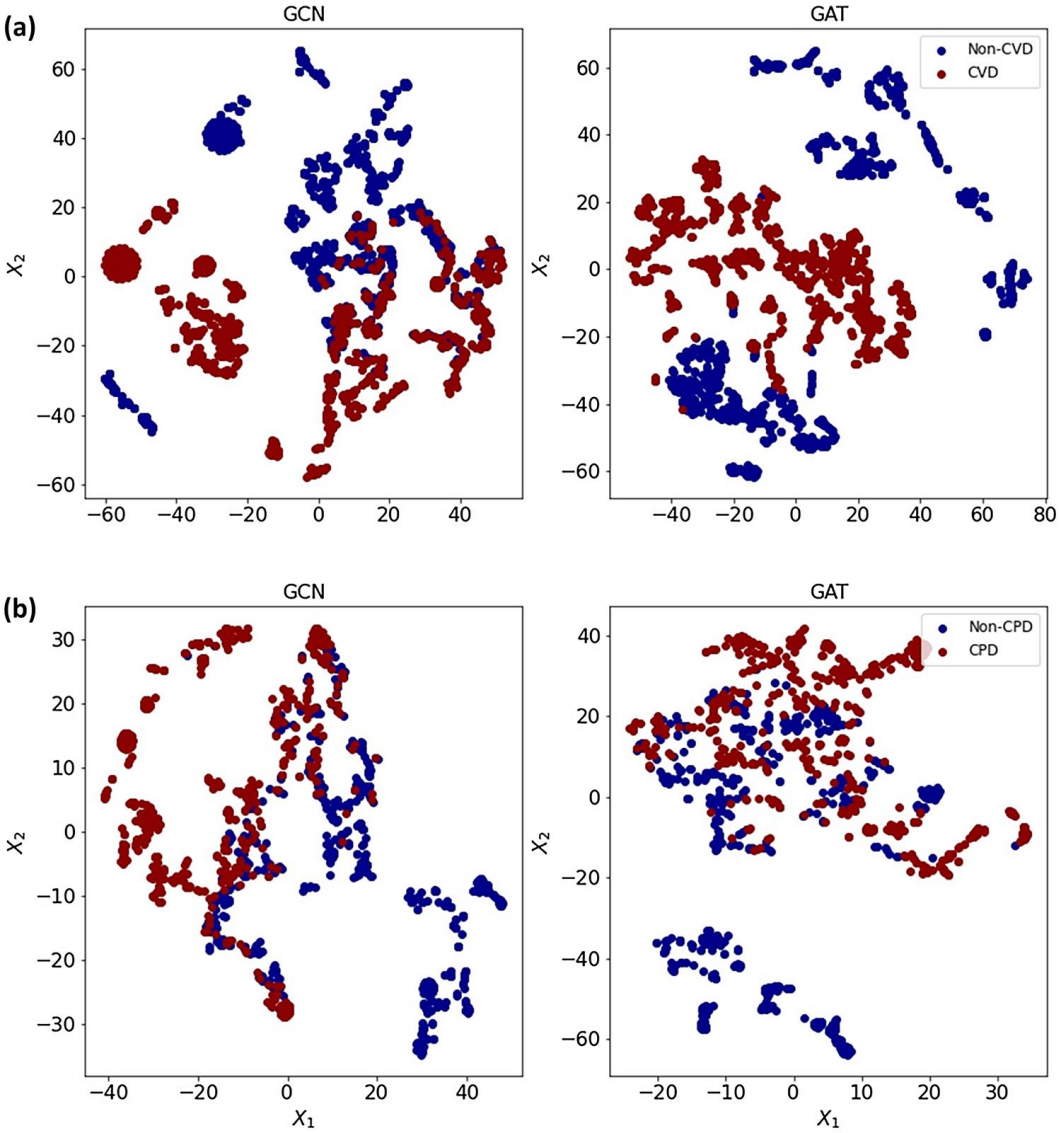
(**a**) t-SNE visualisation of GNN-Based models embeddings for CVD. (**b**) t-SNE visualisation of GNN-Based models embeddings for CPD. Node colours denote labels.

In addition to predicting the risk of chronic disease (node class), it is helpful to get a more detailed picture of what information the GNN-based models have learnt about from patients suffering from common diseases. This means an embedding of the node into a latent vector space that captures that information, which is a neural network that generates those vectors. For GNN-based models, we use the model’s final graph convolution layer before applying the prediction layer. These node embeddings can be seen as points on a graph with their true labels (i.e., CVD or Non-CVD and CPD or Non-CPD). Supposedly, the model has learned relevant information about the nodes based on their class. In this instance, we should anticipate observing clusters of patients in the node embedding space, with patients of the same labels belonging to the same cluster. However, the output dimension of the last GCN layer was 16, implying that each embedding is made up of 16 numbers. At the same time, the embeddings for GAT returned are 64-dimensional features (8 dimensions for each of the 8 attention heads) for all nodes. Directly plotting these points will require a more than two-dimensional plot, which is difficult for humans to comprehend. Alternatively, we may reduce these vectors to two dimensional, resulting in two-dimensional vectors that can be shown on a standard 2D scatter plot using t-Distributed Stochastic Neighbour Embedding (t-SNE)^52^. Figure 3a, b show the t-SNE visualisation of GCN and GAT model embeddings for CVD and CPD, respectively. We can see the two clusters in the t-SNE plot, verifying the GNN-based model’s discriminative power.

## Discussion

Since chronic diseases often have shared risk factors. Early detection of patients with these chronic conditions will aid in their prevention, which is also beneficial to population health and medical decision-making. This study developed a risk prediction framework for chronic diseases using machine learning with graph methodology. In the example, GNN-based models achieved exceptional prediction results for both CVD and CPD cohorts.

To our knowledge, this is the first study to use the WPN and conduct representation learning directly from the graph to predict the risk of chronic diseases. Most previous studies focused on risk factors, such as age, gender, smoking status, systolic blood pressure and body mass index^6,7,53,54^. We verified GNN-based models’ performances by constructing the state-of-the-art machine learning models (i.e., ANN) that solely operates on input node features. ANN performed poorly when compared to the best-performing GAT model, with only 71.84% and 61.79% test accuracy for CVD and CPD, respectively. The fundamental reason for this is that ANN fails to incorporate an essential bias. There are latent relationships between patients if they are diagnosed with the same disease. We proposed a WPN to extract the latent relationship among patients. However, traditional machine learning and classic neural network rely on hand-engineered features and are constrained by their inflexibility^55^. GNN-based models can help to boost performance by capturing the information of graphs. The framework proposed in this study automatically learns the features from the weighted patient network, which is different from the previous similar studies using network features to predict the risk of chronic disease^21^. If new data is added, the existing technique needs to recalculate each patient’s network features, which is computationally expensive. Compared to the previously followed approach, the advantage of this study is that if a new patient is added, there is no need to recalculate the patient’s network features. Instead, the proposed framework learns the network’s features automatically.

We evaluated the framework with network features (i.e., centrality and clustering measures) from the patient network. The inclusion of network features increases the accuracy for baseline models but inconsistent changes for both GCN and GAT. The GCN accuracy increases for CVD with the consideration of network measures in the model but decreases for CPD. The GAT accuracy also decreases for CVD and CPD with the inclusion of network measures in the model. Since the attention mechanism assigns varying importance to each neighbour’s input, it improves learning capacity by utilising the anisotropy paradigm^41^. The network features also capture the importance of nodes. Cumulative aggregation reduces the signal and increases the noise, causing the neural network to train slowly and perform poorly. In addition, there are a large number of ways to express network characteristics^56^. Some latent information may be lost from the previous study because it is impossible to use all network features. From the literature, Duong et al.^57^ used network features on GNN-based models for node classification and graph classification tasks. The features include degree^58^, DeepWalk^59^ and PageRank^60^. The results show that GNNs perform well when node characteristics and node labels have a strong association. Therefore, this may constitute the area of future studies.

We consider edge weight in the patient network and develop the GNN-based disease prediction framework that uses both node features and edge weights. The edge weights affect message aggregation. Inclusion of weight in the patient network increases the accuracy of the GCN model since the weighted adjacency matrix stores the weight of the edges, which reflects the importance of the relationship between patients. Although due to the distribution of data, the improvement in accuracy is not obvious, future research might apply different administrative data to examine this framework.

There are various limitations to this study. The majority of these are related to the limitations of real-world health datasets. For example, the coding quality may range from one hospital to the next and in different periods. In addition, healthcare policy changes regularly, which might have an impact on coding practice. Further, the administrative claim data is a summary of hospital admissions and discharges. As a result, it does not include information about general physician visits and subsequent diagnoses. This could lead to an underestimation of a patient’s comorbidities. Lastly, as this study employs a dataset based on the Australian context, we need to compare the results from this study with administrative data from other countries or other insurance companies to confirm the generalisability of the findings of this study.

## Conclusion

This study developed a novel weighted graph-based framework for chronic disease prediction by constructing a graph from administrative claim data. Firstly, WPN was created from patient-disease bipartite graph projection. Then, taking advantage of GNNs models, the proposed framework captures a variety of latent relationships between patients. The experimental results on a real-world dataset show promising effectiveness of our proposed framework, especially in a model with the attention mechanism.

As a result, this research can assist healthcare providers in making practical use of their data. The framework can be used to provide timely advice or additional treatment suggestions for patients who are at risk of developing chronic diseases.
